# Curriculum mapping of a dental materials science course: a reality check and way forward

**DOI:** 10.1186/s12909-023-04717-z

**Published:** 2023-10-02

**Authors:** Galvin Sim Siang Lin, Jia Yee Foo, Chan Choong Foong

**Affiliations:** 1 Department of Dental Materials, Faculty of Dentistry, Asian Institute of Medicine, Science and Technology (AIMST) University, Bedong, Kedah 08100 Malaysia; 2 Medical Education and Research Development Unit (MERDU), Faculty of Medicine, University of Malaya, Kuala Lumpur, 50603 Malaysia

**Keywords:** Curriculum, Dental education, Dental materials, Pedagogy, Undergraduate

## Abstract

**Background:**

Dental materials science is an important subject, but research on curriculum mapping in preclinical dental materials science courses is still scarce. The present study aimed to conduct a curriculum mapping in analysing elements and suggesting recommendations for an institutional dental materials science course.

**Methods:**

Curriculum mapping was conducted for the Year 2 undergraduate dental materials science course (Bachelor of Dental Surgery programme) in a Malaysian dental school. Based on Harden’s framework, the following steps were used to map the curriculum of the institutional dental materials science course: (1) scoping the task; (2) deciding the mapping format; (3) populating the windows, and (4) establishing the links. Two analysts reviewed the curriculum independently. Their respective analyses were compared, and discrepancies were discussed until reaching a consensus. A SWOT analysis was also conducted to evaluate the strengths, weaknesses, opportunities, and threats associated with the curriculum.

**Results:**

Course learning outcomes, course contents, levels of cognitive and psychomotor competencies, learning opportunities, learning resources, learning locations, assessments, timetable, staff, curriculum management and students’ information were successfully scoped from the institutional dental materials science course. The present curriculum’s strengths included comprehensiveness, alignment with standards, adequate learning opportunities, well-defined assessment methods, and sufficient learning resources. However, the identified weaknesses were repetition in curriculum content, limited emphasis on the psychomotor domain, dependency on a single academic staff, and limited integration of technology. The SWOT analysis highlighted the opportunities for curriculum improvement, such as revising repetitive content, emphasising the psychomotor domain, and incorporating advanced teaching strategies and technology.

**Conclusions:**

The present dental materials science curriculum demonstrated several strengths with some areas for improvement. The findings suggested the need to revise and optimise the course content to address gaps and enhance student learning outcomes. Ongoing monitoring and evaluation are necessary to ensure the curriculum remains aligned with emerging trends and advancements in dental materials science.

## Background

Dental materials science is an important subject in the preclinical years of undergraduate dental curricula that integrates the knowledge of materials science and chemical engineering. Due to its multidisciplinary nature, students may find it challenging to comprehend its fundamental concepts and demonstrate clinical applications of materials science and engineering [1]. Discouragingly, dental materials science is commonly delivered using the didactic method via a series of lectures [2]. The use of such a conventional educational approach not only led to a dearth of application of relevant knowledge, but also reduced students’ enthusiasm and learning effectiveness toward this subject [1, 2]. Moreover, since new biomaterials emerge over time, undergraduate dental curricula must be regularly revised to reflect the ongoing emergence of various dental materials, and to improve delivery methods to enable students to demonstrate clinical applications of the subject.

In Malaysia, dental materials science course is primarily delivered in the second year of most undergraduate dental programmes. The Faculty of Dentistry, Asian Institute of Medicine, Science and Technology (AIMST) University Malaysia is one of the oldest private dental institutes in the country that offers a five-year Bachelor of Dental Surgery (BDS) undergraduate programme. At present, the institution is adopting an outcome-based education model that focuses on the learning outcomes of students. Dental materials science is a core course in the preclinical phase of the BDS programme which is integrated as a part of the restorative dentistry subject, and it is distributed across four modules over the second year of the dental curriculum [3]. Despite being integrated as part of the restorative dentistry subject, the dental materials science course is still introduced as a stand-alone course within the subject, rather than being discipline-based. In view of the Malaysian Qualifications Agency’s recommendation for curriculum review to be carried out every five years to keep abreast with contemporary demands [4], it is a good practice for dental educators to analyse existing curriculum and subsequently identify essential elements that require changes, prior to proposing the changes in the curriculum review [5].

In reviewing and re-designing a curriculum that provides quality education, a series of coherent steps is required [6]. One of the most recognised steps is curriculum mapping. Curriculum mapping is a technique that can be used to identify what is taught, how it is taught, when it is taught, and the assessments used to determine whether the students have attained the desired learning outcomes [7]. A well-crafted curriculum is the culmination of the course materials, learning objectives, instructional strategies, assessment, learning environment, and learning theories adopted [7]. By outlining and correlating these crucial curricular elements, curriculum mapping may offer transparent and authentic teaching and learning structures to be presented to all stakeholders involved, including instructors, students, curriculum developers, general public, and researchers [7]. Curriculum mapping is an effective tool to provide an overall picture of the entire curriculum.

To the best of the authors’ knowledge, curriculum mapping for dental materials science courses is still scarce in the literature. Therefore, the present study aimed to map an institutional dental materials science course, and next, to identify if there is any gap that may be addressed by re-designing a new curriculum. The present study also enabled the verification of curriculum alignment between expected course learning outcomes and current demands in dental education [8].

## Methods

Curriculum mapping for the dental materials science course in Year 2 BDS (Batch 15, Year 2022) was conducted based on Harden’s framework [7]. The following steps were: (1) scoping the task; (2) deciding the mapping format; (3) populating the windows, and (4) establishing the links. The first and second authors independently performed a thorough perusal of the 2022 BDS dental materials science curriculum. Each analyst retrieved and identified relevant course information and documented it in the form of Microsoft Excel spreadsheet. Two spreadsheets were compared, and discrepancies were discussed until a consensus was reached. Subsequently, a SWOT analysis was conducted to assess the strengths, weaknesses, opportunities, and threats associated with the curriculum [9].

### Scoping the task

Harden suggested 10 windows for curriculum mapping, which includes the expected learning outcomes, curriculum content, assessment, learning opportunities, learning location, learning resources, timetable, staff, curriculum management, and student information [7]. In the present study, all 10 windows were included, with the addition of a column listing the level for each topic according to Bloom’s (cognitive) and Simpson’s (psychomotor) taxonomies.

### Deciding the format

A table was used to represent the outcomes of the curriculum mapping. Tables as an illustration tool can hold a significant quantity of data and give a clear overview of the curriculum.

### Populating the windows

Information regarding the course learning outcomes, course content, Bloom’s/Simpson’s taxonomy levels, credit hours, learning opportunities, learning resources, learning location, assessment, academic staff involved, curriculum management, and student information were obtained from the BDS Year 2 Student Handbook. The course timetable is not shown in this article because it would be over-extensive for word counts.

### Establishing the links

Links between different windows were established. Course learning outcomes (CLOs) were mapped with the programme learning outcomes (PLOs) and the curriculum contents. Furthermore, learning opportunities, learning resources, learning locations, and methods of assessments were also identified and linked to ensure that an appropriate learning environment and proper guidance are provided to students. Relationships between these various windows would allow a meticulous inspection of the entire dental materials science curriculum. Last, any curriculum content gaps, repetitions or other flaws were identified.

## Results

### Scoping the task

#### Course learning outcomes (CLOs)

The CLOs for the dental materials science course have been formulated and approved by the Malaysian Qualifications Agency (MQA) in accordance with the Programme Learning Outcomes (PLOs) Standard (Table 1). The first CLO met the first PLO standard (PLO1: Knowledge), whereas the second CLO met the second PLO standard (PLO2: Practical and Clinical Skills).

**Table 1:** Mapping of the PLOs to CLOs of dental materials science course based on Bloom’s/Simpson’s taxonomy levels

Programme Learning Outcomes		Course Learning Outcomes	Bloom’s (C) / Simpson’s (P) Levels
1.	Apply the scientific knowledge to support, safe, effective, and efficient oral health care.	Explain the properties, requirements, composition, setting reaction, indications, contraindications, manipulative variables, and applications of dental materials	C2
2.	Perform independent general dental practice safely and effectively with good practical and clinical competency.	Demonstrate the practical skills required in simple and complex restorative procedures and fabrication of removable prosthesis with the appropriate handling and manipulation of dental materials	P4
3.	Demonstrate social skills and to display social responsibility involving the patient and the community in decisions relating to their health.		
4.	Display good behaviour and apply ethical values as outlined by the dental profession and abide by the laws governing professional dental practice.		
5.	Discuss effectively with peers in the dental and other health profession, patients, and community; develop teamwork and exhibit leadership qualities.		
6.	Analyse critically and use latest scientific knowledge and techniques to solve problems for the benefit of patient.		
7.	Practice principles of lifelong learning and participate in continuing professional development activities.		
8.	Organise and manage general dental practice clinic and community programme with entrepreneurial spirit and skills to improve the oral health of the public		

#### Course contents

The dental material science course was divided into four modules comprising 25 lectures and 3 practical classes, which covered both clinical and laboratory-based dental materials.

#### Bloom’s and simpson’s levels of taxonomy

The six cognitive levels in Bloom’s taxonomy are knowledge (C1), comprehension (C2), application (C3), analysis (C4), synthesis (C5), and evaluation (C6) [10]; while the Simpson’s psychomotor domain is divided into seven levels: perception (P1), set (P2), guided response (P3), mechanism (P4), complex or overt response (P5), adaptation (P6), and origination (P7) [11]. Based on the dental materials science course, all course materials were deemed to be at the C1 or C2 cognitive levels, and P4 psychomotor level. Nevertheless, most of the curriculum content (82%) was cognitive in nature, with only 18% accounting for psychomotor domains.

#### Learning opportunities

Most of the faculty-provided learning opportunities included lectures, practical classes, and self-learning sessions. Students were given the opportunity to participate in practical classes to develop their skills and grasp the properties of various dental materials.

#### Learning resources

Resources available to aid the students’ learning included textbooks, printed notes, and demonstrations.

#### Learning locations

Settings where the students’ learning activities occur are referred to as learning locations. Practical classes in the present curriculum were conducted in the simulation lab, while lectures on dental materials science were held in the lecture halls.

#### Assessments

Students’ assessments were organised into three parts: continuous assessments, final examination, and viva voce. Continuous assessments aimed to evaluate the students’ cognitive and psychomotor skills throughout the course. These covered theory-written tests, lab practical assessments, and seminar presentations. Multiple-choice questions (MCQ), and short answer questions (SAQ) were included in the theory tests, along with the objective structured practical assessments (OSPA) which were administered at the end of the first three modules. Continuous assessments contributed 40% to the final grade. On the other hand, the final examination contributed to 60% of the final grade, and it consisted of theory written examination, OSPAs, and a viva voce. Students’ final grades were determined by summing continuous assessments and final examination.

#### Timetable

The timetable was arranged by the Year 2 coordinator to synchronise with the flow of course contents from Module 1 to Module 4. Student learning time (SLT) was allocated based on the weightage and complexity of each topic in the syllabus. The total SLT in the dental materials science course was approximately 135 credit hours.

#### Staffs

All dental materials science course content was taught by one academic staff in the Department of Dental Materials who has a postgraduate degree in the related field.

#### Curriculum management

The same academic staff who teaches the course acted as the subject coordinator. All educational activities and funding were managed by the subject coordinator.

#### Students’ information

Student prerequisite information was included in the curriculum in which students must pass the Year 1 BDS programme in order to enrol in the Year 2 dental materials science course.

### Deciding the format and populating the windows

The dental materials science course for BDS Year 2 was mapped and presented in Table 2. Table format was chosen because elements in the curriculum can be displayed in a form that is user-friendly for students and academic staff.

**Table 2:** Mapping of the dental materials science course based on Harden’s Framework

Module	No	Course Content	CLO	Bloom’s (C) / Simpson’s (P) Levels	Credit Hours	Learning Opportunities	Learning Locations	Learning Resources	Assessments	
									**Continuous Assessments**	**Final Examination**
1	1.	Properties of Dental Materials	CLO1	C1, C2	135	Lectures Self-learning	Lecture hall	Lecturers Textbooks Printed notes Live demo	Written test (MCQ + SAQ) OSPA Seminar presentations and assignments Lab practical assessments	Written exam (MCQ + SAQ) OSPA Viva voce
	2.	Gypsum Products for Dental Casts and Waxes		C1, C2						
	3.	Impression Materials		C1, C2						
	4.	Introduction to Direct Filling Materials		C1, C2						
	5.	Synthetic Polymers		C1, C2						
	6.	Denture Base Polymers		C1, C2						
	7.	Bonding of Resin-based Materials		C1, C2						
2	1.	Resin-based Filling Materials	CLO1	C1, C2		Lectures Practical classes Self-learning	Lecture hall Simulation lab Dental technology lab			
	2.	Glass Ionomer Restorative Materials		C1, C2						
	3.	Resin-Modified Glass Ionomers		C1						
	4.	Advances in Tooth-coloured Restorative Materials		C1, C2						
	5.	Dental Amalgam		C1, C2						
	6.	Requirements of Dental Cements for Lining, Base and Luting Applications		C1, C2						
	7.	Denture Lining Materials		C1, C2						
	8.	*Practical*: Manipulation of Dental Materials	CLO6	P4						
3	1.	Metals and Alloys	CLO1	C1, C2		Lectures Practical classes Self-learning	Lecture hall Simulation lab Dental technology lab			
	2.	Gold and Alloys of Noble Metals		C2						
	3.	Base Metal Casting Alloys		C1, C2						
	4.	Casting Techniques		C1, C2						
	5.	Investments and Refractory Dies		C1, C2						
	6.	Steel and Wrought Alloys		C1, C2						
	7.	*Practical*: Mechanical Properties	CLO6	P4						
4	1.	Impression Materials	CLO1	C1, C2		Lectures Practical classes Self-learning	Lecture hall Simulation lab			
	2.	Ceramics		C1, C2						
	3.	Porcelain Fused to Metal (PFM)		C1, C2						
	4.	*Practical*: Viscoelastic properties	CLO6	P4						

### Establishing the links

Following the curriculum mapping, it is evident that the curriculum content has addressed the course learning outcomes, with CLO1 being addressed by all lecture-based curriculum content and CLO6 being covered by practical content. Nevertheless, the mapping has revealed that there is a repetition in the lecture topic ‘*Impression Materials*’. The lecture topic is taught twice in Module 1 and 4, respectively, with similar learning objectives. Most of the curriculum content accommodated the learning outcomes at both the C1 and C2 cognitive levels, with the lecture topic ‘*Resin-modified Glass Ionomers*’ covering only C1 cognitive level and ‘*Gold and Alloys of Noble Metals*’ covering only C2 cognitive level. The P4 psychomotor learning outcomes have been included in the practical classes.

All curriculum content was complemented by appropriate learning opportunities, which include lectures, self-learning sessions, and practical classes. The lecture hall was used for lectures, and the simulation lab was used for practical classes. In addition, there were sufficient resources available to support student learning, such as academic staff from the dental materials science department, textbooks, printed notes, and demonstration of mixing and manipulating different dental materials during practical activities. Both continuous assessments and final examination were appropriate for the cognitive and psychomotor levels of the course content.

### SWOT analysis

**Fig. 1:**
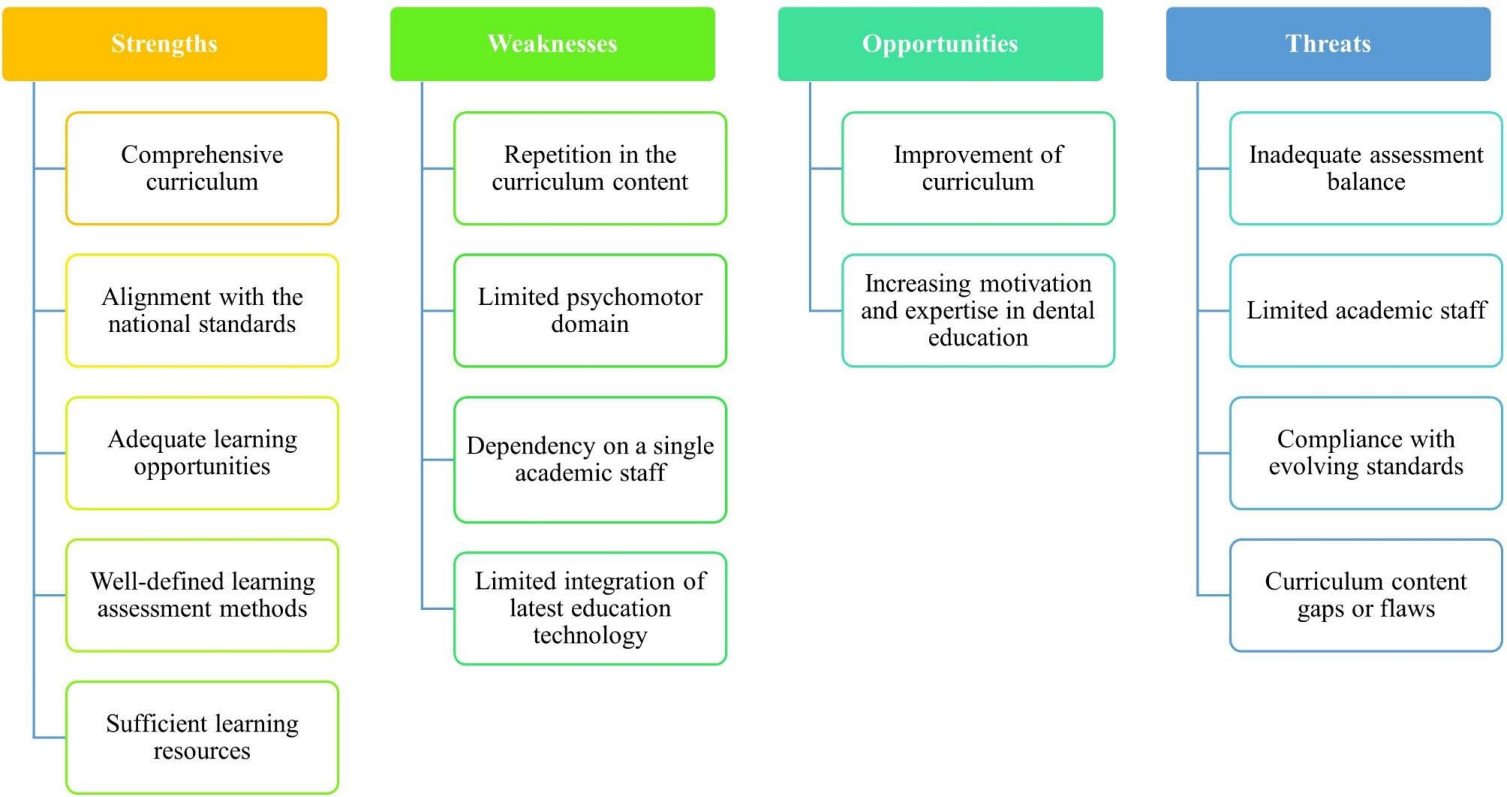
SWOT analysis of the dental materials science course

The strengths, weaknesses, opportunities, and threats of the dental materials science course were analysed (Fig. 1).

#### Strengths (what goes well in the curriculum)

1. Comprehensive curriculum: The dental materials science curriculum covered a comprehensive list of relevant topics. It also included various elements for the curriculum such as learning outcomes, course content, assessments, learning opportunities, and resources.
2. Alignment with the national standards: The course learning outcomes (CLOs) were formulated and approved by the Malaysian Qualifications Agency (MQA) in accordance with the Programme Learning Outcomes (PLOs). This ensures that the course has clear objectives and aligns with the overall programme goals.
3. Adequate learning opportunities: There were various learning opportunities, including lectures, practical classes, and self-learning sessions. This allows students to acquire both theoretical knowledge and practical skills related to dental materials science.
4. Well-defined assessment methods: The assessments were organised into three parts: continuous assessments, final examination, and viva voce. They evaluated students’ cognitive and psychomotor skills throughout the course and ensured a valid and reliable evaluation of their knowledge and abilities.
5. Sufficient learning resources: Students had access to resources such as textbooks, printed notes, and demonstrations, which support their learning and understanding of the dental materials science course.

#### Weaknesses (what is lacking in the curriculum)

1. Repetition in curriculum content: The curriculum mapping revealed a repetition in the lecture topic *‘Impression Materials*’, which was taught twice in Module 1 and Module 4 with similar learning objectives. The identification of repetitive lecture topics and the variation in cognitive levels (C1 and C2) presented an opportunity to refine and optimise the course content, ensuring that all learning outcomes will be addressed effectively.
2. Limited psychomotor domain: The curriculum mapping showed that only 18% of the curriculum accounted for the psychomotor domain, while the majority (82%) focused on the cognitive aspect. This imbalance may have an impact on the development of sufficient practical skills and hands-on experience for students. Students would benefit from increasing the emphasis on the psychomotor domain, hands-on experiences, and practical activities.
3. Dependency on a single academic staff: The dental materials science course was taught by one academic staff who also acted as the subject coordinator. While this may provide consistency, it could limit the diversity of teaching styles, perspectives, and expertise available to students.
4. Limited integration of the latest education technology: The curriculum could consider integrating technology, such as virtual simulations or interactive learning tools, to enhance student engagement and understanding of the content.

#### Opportunities (how the curriculum can be improved)

1. Improvement of curriculum: Repetitive lecture content and the variation in cognitive levels (C1 and C2) can be revised. More practical skills should be incorporated into the curriculum to increase the emphasis on the psychomotor domain. Furthermore, the implementation of new pedagogical strategies with advanced technology can be further explored.
2. Increasing motivation and expertise in dental education: Some growing initiatives, including issues and solutions to the contemporary dental curriculum, are widely discussed in conferences and journals. Thus, dedicated dental educators can pursue academic degrees in dental or health professions education, and establish dental education departments. These initiatives will contribute to the formation of a group of dental educators who are motivated to revise dental education for betterment.

#### Threats (what are the obstacles)

1. Inadequate assessment balance: Corresponding to the need for more emphasis in psychomotor domain, the weightage of continuous assessments (40%) and the final examination (60%) may not provide an ideal balance for evaluating both cognitive and psychomotor skills. However, the revamping of assessment methods for the entire dental curriculum may be beyond the control of the individual academic staff.
2. Limited academic staff: The dental materials science course was taught by a single academic staff member. This reliance on a single staff member may pose a challenge in terms of workload and potential limitations in providing diverse perspectives and expertise. There is also a possibility that the institution might not be able to recruit more academic staff after considering costs and revenues.
3. Compliance with evolving standards: The present curriculum mapping project is aligned with the existing MQA standards, but changes in programme standards and regulations may require regular updates and revisions. Curriculum mapping is needed to ensure constant compliance with the national standards. Meanwhile, different national and international regulatory (or recognition) bodies might have different standards.
4. Curriculum content gaps or flaws: Although efforts have been made to identify and address any curriculum content gaps, there is a possibility of undiscovered gaps or flaws. Ongoing monitoring and evaluation are needed to ensure the curriculum remains up to date and aligned with emerging trends and advancements in dental materials science.

## Discussion

The present study conducted a curriculum mapping for the dental materials science course in Year 2 of the BDS programme. The Malaysian Dental Deans’ Council has proposed a new competencies framework for dental graduates, which is in alignment with the Malaysian Qualification Framework (MQF) version 2.0 [12]. One of the newly added competencies which expect graduates to be able to justify the selection of various dental materials (C5 of Bloom’s taxonomy level), and it implies that dental students must attain a higher-order cognitive ability in the dental materials science course. Therefore, a lower-order cognitive ability might pose a serious threat in a programme which is supposed to produce competent dental practitioners, despite some might see it as an “overwhelming” appeal to include higher-order cognitive ability in the preclinical undergraduate dental curriculum. Competencies of dental practitioners who graduated from an ill-structured curriculum may be called into question. Dentistry is a profession that primarily emphasises substantial hands-on activities and a high degree of cognitive abilities. Consequently, different Malaysian dental institutions should establish a national curriculum for the dental materials sciences course, in accordance with the new competencies’ framework.

The present study employs Harden’s framework to map and evaluate its institutional dental materials science course for the second year BDS programme. This comprehensive approach ensures that all relevant aspects of the curriculum are considered. Based on the curriculum mapping results, the course is cognitively oriented, rather than accommodating psychomotor. The results rear the question of whether more practical classes are necessary as students would need to be able to demonstrate practical and manipulation skills for different dental materials. Some may argue the need for evidence supporting the notion that practical classes can improve students’ performances in dental materials science courses, but it has been reported that practical training can enhance students’ awareness of the importance of procedures and boost their self-confidence in managing patients [13]. It is also worth noting that dental students score better in subjects that incorporate practical exercises [14]. Hence, the present study postulates that incorporating more practical exercises in mixing and manipulating various types of dental materials, it can enhance educational experiences and outcomes.

Next, based on the curriculum mapping results, the is a lack of variety of teaching strategies in the institutional dental materials science course. Learning topics are often delivered in a one-way approach via didactic lectures, with only three practical classes to support students’ practical experiences. To revamp conventional teaching strategies, the literature suggests that several cutting-edge strategies could improve students’ engagement and academic performance in dental materials science, such as adopting flipped classrooms [15], modified microteaching [1], crossword puzzles [16], jigsaw learning [17], and case-oriented small group learning [14]. Based on previously published studies, researchers concluded that the use of flipped classrooms and modified microteaching significantly raised students’ satisfaction and learning interests, improved teaching effectiveness, enhanced critical thinking skills, and allowed higher availability of subject material [1, 15]. Furthermore, other innovative pedagogical strategies (crossword puzzle, jigsaw learning, case-oriented small group learning, or peer training) were reported to facilitate active learning, reduce classroom tension, and improve students’ confidence in clinical practice [14, 16–18].

In addition, curriculum integration has become a point of concern following the curriculum mapping results. The traditional dental curriculum has fractionated its subjects into preclinical and clinical phases. In the preclinical phases, the institutional dental materials science course tends to confine students’ learning to acquiring factual knowledge, without emphasis on clinical application. Subsequently, it is challenging for the students to effectively apply theoretical knowledge in their clinical practice. Therefore, an 11-step integration ladder for health professions curriculum, which moves away from subject-based teaching to inter- and trans-disciplinary integrated teaching, may be able to facilitate students’ clinical application of theoretical knowledge [19]. When comparing the institutional dental materials science course with the other courses in the undergraduate dental programme, it can be inferred that existing teaching strategies are oriented towards the lower part of the integration ladder. In other words, other courses which are relevant to dental materials science are sparsely correlated, and teaching strategies of the other courses are designed in silos.

A greater level of horizontal and vertical integrations between various courses is advocated to improve learning efficacy, and to prepare for the high-fidelity circumstances that students would be encountering in clinical scenarios [20]. Horizontal integration involves establishing the relationship within multiple dental courses [21]. For example, properties of restorative materials in the dental materials science course are related to tooth preparation techniques in conservative dentistry and the choice of prostheses in prosthodontics. In addition, topics like dental stem cells, tissue engineering, regenerative dentistry, and nanotechnologies may be incorporated into the undergraduate dental curriculum and related to the field of fundamental medical sciences, including biochemistry and biomedical science [3]. With this horizontal integration method, students are able to connect the basic ideas of dentistry to those in bioengineering and material sciences.

Meanwhile, vertical integration implies incorporating knowledge and skills from an early phase (e.g., year 1) to the latter and advanced phases within the five-year BDS programme. The theoretical aspects of dental materials science content such as the basic properties and chemical compositions of dental materials can be covered in the preclinical year of the BDS programme, while clinically relevant content related to applied dental material science should be covered in the clinical phase of the curriculum [3]. For instance, topics such as ceramics and metal-ceramic materials can be revisited in the latter phases of the programme when students are exposed to fixed prosthodontics. By covering the theoretical aspects initially, students can develop a solid foundation, enabling them to better comprehend the practical applications and challenges of dental materials during the clinical phase. This approach ensures a comprehensive understanding of dental materials and their clinical significance, preparing students to make informed decisions regarding material selection, application techniques, and patient management in their future dental practice. Last, curriculum mapping identifies potential gaps in institutional dental materials science course. For instance, the topic “*Impression Materials*” is discovered to recur in the first and fourth modules, necessitating a revision to remove duplicated content.

Assessments are crucial in dental education as they help improve conceptual comprehension, information retention, and the cultivation of critical thinking abilities among students [22]. In the institutional dental materials science course, MCQ and SAQ are employed to evaluate students’ cognitive learning, while OSPA is used to test students’ psychomotor performance. Although group projects, assignments, and seminar presentations are employed as parts of the student assessments, these assessment methods are given little weightage. This imbalance could impact the overall assessment validity and the ability to gauge students’ comprehensive understanding and competency. The dental profession requires practitioners to be competent in cognitive, psychomotor, and affective domains. Hence, introducing holistic assessments such as peer- and self-assessments, or direct observation of procedural skills may comprehensively evaluate a student’s competency across all three domains [23]. The curriculum mapping process also provided insights into students’ prerequisite information, highlighting the requirement for students to pass the Year 1 BDS programme to enrol in the dental materials science course. This prerequisite ensures that students possess the foundational knowledge necessary for successful engagement with the subject matter.

In the present study, a SWOT analysis was conducted after mapping out the curriculum using Harden’s framework. SWOT analysis allows educators and curriculum developers to gain a comprehensive understanding of the curriculum’s current state and identify areas for improvement [9]. For instance, clear learning outcomes is one of the strengths of the present dental materials science curriculum whereby educators can capitalise on this aspect to enhance the overall quality of education. They can build upon the existing learning outcomes and reinforce successful pedagogical strategies and approaches [24]. Meanwhile, the weaknesses identified through SWOT analysis shed light on areas that required improvement or attention. For example, the imbalance between the cognitive and psychomotor domains in the dental materials science curriculum highlights the need to enhance practical skill development by providing more hands-on experiences and practical activities. [25]. In the case of the dental materials science curriculum, opportunities may include integrating technology or adopting innovative teaching methods to enhance student engagement and understanding. Furthermore, the SWOT analysis highlighted potential threats in the present study that may hinder the success of the curriculum. For instance, limited academic staff can pose challenges in terms of workload and expertise [26]. Recognising these threats enables educators to find appropriate solutions, such as faculty development programmes or collaboration with external experts [27], to mitigate the impact of these threats.

While the curriculum mapping results reveal gaps and recommend improvements for dental materials science courses, the redesign of the courses should consider an appropriate instructional design. Instructional design refers to the systematic process of developing effective and efficient instruction [28]. The basis for its effectiveness and efficiency lies in the evidence used in the analysis, design, development, implementation, and evaluation of instruction [29]. Before redesigning and redeveloping the course objectives, evidence should be collected from analysing and validating the actual performance (weaknesses), desired performance (expectations), and reasons for the weaknesses [29]. Evidence-based instructional design also includes the application of educational psychology to justify teaching and learning strategies for courses, in contrast to intuition [30]. Meanwhile, pilot testing or formative evaluation of courses produces feedback (evidence) when deciding whether the existing design can be continued, or a revision is needed. In short, the redesign of dental materials science courses should be evidence-driven.

Nonetheless, the present study has limitations. It is an institutional work and wishes to stimulate a national effort to operationalise curriculum mapping at different local institutions. Next, these curriculum mapping results could be compared in identifying strengths and gaps. This collective effect shall delve into establishing a national curriculum for dental materials science courses, in alignment with the new competencies’ framework for dental graduates.

## Conclusion

The present study represents a Malaysian initiative to demonstrate the values of curriculum mapping for the dental materials science course. Curriculum mapping is a feasible and transparent method for identifying curriculum elements, revealing gaps, and recommending improvements. Based on the mapping results, the strengths of the curriculum included its comprehensiveness, alignment with standards, adequate learning opportunities, well-defined assessment methods, and sufficient learning resources. However, the identified weaknesses included repetition in curriculum content, limited emphasis on the psychomotor domain, dependency on a single academic staff, and limited integration of technology. Horizontal and vertical curricular integration, as well as adopting cutting-edge teaching strategies and holistic assessments are recommended to improve the course. Nevertheless, the present emphasised the importance of regularly reviewing and revising the dental materials science curriculum to ensure its alignment with contemporary demands and to enhance students’ learning experiences. By addressing the identified weaknesses and leveraging the opportunities, dental educators can optimise the curriculum to better prepare students for the dental profession. Furthermore, ongoing monitoring and evaluation are necessary to ensure the curriculum remains up to date and responsive to emerging trends and advancements in dental materials science. Overall, curriculum mapping serves as an effective tool to enhance educational outcomes for dental students.

## Data Availability

The data that support the findings of this study are available from AIMST University Malaysia, but restrictions apply to the availability of these data, which were used under license for the current study, and so are not publicly available. Data are however available from the corresponding author upon reasonable request and with permission of AIMST University Malaysia.

## Abbreviations

AIMST: Asian Institute of Medicine, Science and Technology
BDS: Bachelor of Dental Surgery
CLO: Course Learning Outcome
MCQ: Multiple-choice questions
MQA: Malaysian Qualifications Agency
MQF: Malaysian Qualification Framework
OSPA: Objective structured practical assessments
PLO: Programme Learning Outcome
SAQ: Short answer questions
SLT: Student Learning Time
SWOT: Strengths, Weaknesses, Opportunities, and Threats
